# The Halophyte *Halostachys caspica* AP2/ERF Transcription Factor HcTOE3 Positively Regulates Freezing Tolerance in *Arabidopsis*

**DOI:** 10.3389/fpls.2021.638788

**Published:** 2021-05-13

**Authors:** Fangliu Yin, Youling Zeng, Jieyun Ji, Pengju Wang, Yufang Zhang, Wenhui Li

**Affiliations:** Xinjiang Key Laboratory of Biological Resources and Genetic Engineering, College of Life Science and Technology, Xinjiang University, Urumqi, China

**Keywords:** *Halostachys caspica*, HcTOE3, AP2/ERF transcription factor, transgenic *Arabidopsis*, freezing tolerance

## Abstract

The APETALA2 (AP2) and ethylene-responsive element-binding factor (ERF) gene family is one of the largest plant-specific transcription factor gene families, which plays a critical role in plant development and evolution, as well as response to various stresses. *The TARGET OF EAT3* (*TOE3*) gene is derived from *Halostachys caspica* and belongs to the AP2 subfamily with two AP2 DNA-binding domains. Currently, AP2 family mainly plays crucial roles in plant growth and evolution, yet there are few reports about the role of AP2 in abiotic stress tolerance. Here, we report *HcTOE3*, a new cold-regulated transcription factor gene, which has an important contribution to freezing tolerance. The main results showed that the expression of *HcTOE3* in the *H. caspica* assimilating branches was strongly induced by different abiotic stresses, including high salinity, drought, and extreme temperature (heat, chilling, and freezing), as well as abscisic acid and methyl viologen treatments. Overexpressing *HcTOE3* gene (OE) induced transgenic *Arabidopsis* plant tolerance to freezing stress. Under freezing treatment, the OE lines showed lower content of malondialdehyde and electrolyte leakage and less accumulation of reactive oxygen species compared with the wild type. However, the survival rates, antioxidant enzyme activities, and contents of osmotic adjustment substance proline were enhanced in transgenic plants. Additionally, the OE lines increased freezing tolerance by up-regulating the transcription level of cold responsive genes (*CBF1*, *CBF2*, *COR15*, *COR47*, *KIN1*, and *RD29A*) and abscisic acid signal transduction pathway genes (*ABI1*, *ABI2*, *ABI5*, and *RAB18*). Our results suggested that HcTOE3 positively regulated freezing stress and has a great potential as a candidate gene to improve plant freezing tolerance.

## Introduction

Cold temperature is an important circumstance stress element, which restricts the plant growth, evolution, yield, and geographic dissemination, and it is a kind of natural disaster often encountered in agricultural production (Ding et al., 2019). Low-temperature stress impacted the plant cell membrane and the activities of reactive oxygen species (ROS)–scavenging enzymes, as well as protein stability (Orvar et al., 2000). These processes led to photoinhibition and impaired photosynthesis and caused severe membrane destruction (Siddiqui and Cavicchioli, 2006; Ruelland et al., 2009). According to the different degrees of cold temperatures, there are two major categories of plant damage: chilling injury (greater than 0°C) and freezing stress (less than 0°C) (Liu et al., 2018). Freezing stress not only caused chilling injury, but also generated more damage to affected plants, including cell and plant death (Pearce, 2001).

During long-history evolution process of plants, they have evolved different mechanisms for handling low-temperature stress, including activating ICE1–CBF–COR signaling pathway (Thomashow, 1999). The expression of *CBF* genes was quickly up-regulated by cold treatment, and further induced the overexpression of *COR* genes, thereby improving plant tolerance to cold stress (Gilmour et al., 1998). In addition, there is also an abscisic acid (ABA)–dependent cold signaling pathway. The expression of some cold temperature–related genes was regulated and induced by ABA (such as *RAB18*, *RD29b*) (Uno et al., 2000). Their promoters of these genes contained ABA response elements (ABREs), which initiated a series of plant responses to resist low temperature and enhanced plant cold tolerance (Agarwal and Jha, 2010). Under cold stress conditions, plants also synthesized certain cold-responsive proteins and small molecules, such as proline and soluble sugars for enhancing plant physiological response to cold stress (Kaplan and Guy, 2004; Kaplan et al., 2007). These cold-responsive proteins and small molecules were involved in regulation of cell membrane stability and osmotic potentials, as well as removing certain ROS during plant response to cold stress (Lee et al., 2002; Doherty et al., 2009).

Transcription factors (TFs) are an extensive class of proteins, which play important functions in regulating plant growth and development, as well as responding to various stresses (Xu et al., 2011; Mizoi et al., 2012). The APETALA2 (AP2) and ethylene-responsive element-binding factor (ERF) gene family is one of the largest plant-specific TF gene families. According to the number and binding sequence of AP2/ERF DNA-binding domain (BD), the AP2/ERF TF family was categorized into five subfamilies: AP2, ERF, DREB RAV, and Soloist (Nakano et al., 2006; Cao et al., 2020). Each subfamily contains different domain that can bind to different DNA sequence (Nakano et al., 2006; Cao et al., 2020).

There have been many reports on AP2/ERF genes conferring plant tolerance to abiotic stresses. *BpERF13* was cloned from *Betula platyphylla*, and overexpressing of *BpERF13* gene enhanced plant tolerance to cold stress in transgenic *B. platyphylla* (Lv et al., 2020). The *DcDREB1A* gene was isolated from carrots, and ectopic expression of *DcDREB1* increased plant tolerance to drought in *Arabidopsis* (Li et al., 2020). Overexpressing of *ERF1-V* from *Haynaldia villosa* improved wheat resistance to fungal disease, and the transgenic wheat with *ERF1-V* also induced plant tolerance to salt and drought stresses (Xing et al., 2017). *CmRAV1*, a new RAV gene, was isolated from melon; the heterologous expression of *CmRAV1* enhanced salt tolerance in transgenic *Arabidopsis* during seed germination and early developmental stages (Zhao et al., 2019). However, at present, members of the AP2 subfamily majorly play more functions in affecting floral meristem and determining the identity development of floral organs (Jofuku et al., 1994; Krogan et al., 2012). Additionally, AP2 also functions in maintaining the stem cell niche of bud meristem (Wurschum et al., 2006). Overexpression of *AfAP2-2* gene from *Aechmea fasciata* reduced seed size and delayed flowering in transgenic *Arabidopsis* (Lei et al., 2019).

The halophyte *Halostachys caspica* belongs to Chenopodiaceae. It grows naturally in the semi-desert regions of Central Asia, where saline and alkaline are commonly observed. A previous study showed that this plant species had high tolerance to salt stress (Zeng et al., 2015). The TF TOE3 (*TARGET OF EAT3*) contains two AP2 domains and belongs to the AP2 subfamily. The AP2 subfamily mainly functions in plant growth and development; however, AP2 TF may be also involved in the regulation of abiotic stress tolerance. In our previous work, we isolated and cloned *HcTOE3* gene from *H. caspica*. In this study, we comprehensively identified *HcTOE3*, a new cold stress–responsive *AP2* gene, can improve freezing tolerance in *Arabidopsis* by ectopic expression. Our data demonstrated that HcTOE3 is a positive regulator in enhancing freezing tolerance of plants.

## Materials and Methods

### *HcTOE3* Sequence Identification and Bioinformatics Analysis

The gene and protein sequences of *Arabidopsis* AP2/ERF were downloaded from the PlantTFDB (Jin et al., 2017). PlantTFDB is a publicly available plant TF database. DNAMAN software was employed to construct a multiple amino-acid alignment of the AP2/ERF TFs, including the *H. caspica* TOE3 protein. MEGA7 was employed to construct a phylogenetic tree by the default neighbor-joining method with 1,000 bootstraps. NCBI and SMART^1^ online tools were employed to analyze the existence of the expected AP2 domains in amino acid (aa) sequences. The nuclear localization sequences of HcTOE3 were predicted by using online software^2^. All tested aa sequences can be found in Supplementary Table 1.

### Transcriptional Activation Assay of HcTOE3 in Yeast Cells

*HcTOE3* gene was first isolated and cloned into pGBKT7 plasmid. The primers used in the cloning process can be found in the Supplementary Table 2. Then, the constructed pGBKT7-*HcTOE3* vector was transformed into Y2H yeast cells. At the same time, empty pGBKT7 and pGBKT7-*ScABI3* vectors (Zhang et al., 2018) were also transformed into Y2H yeast cells, which were served as negative and positive controls for transactivation assays. The transactivation activity of each protein was assessed by comparing cell growth on selective medium with SD/-Trp and SD/-Trp-His; the activity was also assessed by the activity of X-α-Gal.

### Subcellular Localization of HcTOE3

pCAMBIA1304-*HcTOE3*-*GFP* vector was constructed by inserting the *HcTOE3* open reading frame cDNA without the stop codon into the plant binary expression vector pCAMBIA1304. Both the constructed fusion vector (*HcTOE3*-*GFP*) and *GFP* vector (serving as negative control) were, respectively, introduced into *Agrobacterium tumefaciens* strain GV3101. Then, the transformed bacteria were used for transient expression analysis in onion epidermal cells (Xu et al., 2014). After 24 h of culture on MS medium, the cells were observed under Zeiss LSM800 laser scanning confocal microscope (Jena, Germany).

### *H. caspica* Culture and Stress Treatments

The seeds of *H. caspica* were collected from the field, located on the edge of Gurbantunggut Desert. The field is extremely saline-alkaline and also considered as a semidesert area in Xinjiang, China. The healthy seeds were cultivated in flowerpots with substrate (perlite: vermiculite: flower soil = 1: 1: 3) and cultured under natural light and temperature (25–28°C). After 8 weeks of growth, *H. caspica* plants were used for profiling the expression of *HcTOE3* gene under 600 mM NaCl, 1,000 mM mannitol, 45°C high temperature, 100 μM methyl viologen (MV), 4°C chilling injury, 0°C freezing point, −2°C freezing stress, and 300 μM ABA treatments, and those seedlings were only irrigated with water as the controls. For each treatment, the assimilating branches were harvested at 0, 3, and 24 h of treatments, respectively. The samples were instantly frozen in liquid nitrogen. Finally, the samples were stored at −80°C until RNA extraction and gene expression profiling.

### Detection of Gene Expression by Quantitative Real-Time Polymerase Chain Reaction

RNAs were isolated from 100 mg sample using Plant RNA Kit (Omega BioTek, United States) by following the instruction provided in the kit. After treatment with DNase I, the RNAs were reversely transcribed into cDNA using SuperScript^TM^ II reverse transcriptase (Takara, Japan) and a poly(dT)_18_ primer. An ABI Prism 7500 Fast Sequence Detection System (Applied Biosystems, United States) was employed to detect the changes in gene expression after stress treatments. A Power SYBR^®^ Green polymerase chain reaction (PCR) Master Mix (Applied Biosystems, United States) was used in the reaction. *HcUBC10* and *Atactin2* were used as the reference genes in these experiments (Shi et al., 2012; Zeng et al., 2015). The relative gene expression level was measured by using the 2^–ΔΔCT^ comparative method (Livak and Schmittgen, 2001). The information of all genes in the quantitative reverse transcription (qRT)–PCR experiments was listed in the Supplementary Table 2.

### Generating Transgenic Plants and the Freezing Treatments

Floral dip method (Clough and Bent, 1998) was employed to transfer *HcTOE3* gene into *Arabidopsis* wild-type (WT) Colombia-0. After harvesting the seeds, all seeds were grown on MS medium containing kanamycin to select the transgenic plants. The selected transgenic plants were verified by the integration and expression analysis of *HcTOE3* gene using genomic PCR and qRT-PCR.

*Arabidopsis* were grown in a greenhouse according to a previous report (Hu et al., 2012). For freezing treatment, plants grown for 4 weeks in the greenhouse were put in a light incubator at −2°C for 60 h and then removed to the greenhouse for continuous growth. At the same time, the phenotypes of each plant were carefully monitored at each treatment stage. The survival rates of each transgenic line and the WT were counted with 46 biological replicates. Among them, the *Arabidopsis* leaf samples were collected for further physiological experiments, histochemical staining, and gene expression at 24 h after freezing treatment.

### Measurement of Physiological and Biochemical Indexes

Membrane permeability was measured according to a previous report (Chen and Wang, 2006). Each repeated collection of 0.1 g leaves was dipped into distilled water for 24 h and then boiled. The conductivity meter (DSS-307, Shanghai, China) was used to measure the initial and postboiling conductivity to calculate the relative conductivity. The contents of malondialdehyde (MDA) and proline were detected. The activities of peroxidase (POD), superoxide dismutase (SOD), or catalase (CAT) were measured by following the manufacturer’s instructions (Beijing Solarbio Technology Co., Ltd., China). Briefly, 0.1 g of tissues was collected and grounded for each sample. The contents of superoxide radical (O${}_{2}^{-}$) and hydrogen peroxide (H_2_O_2_) were detected using NBT and DAB staining method (Shi et al., 2010). The cell damage degree was detected using Evans blue and trypan blue staining. DAB (1 mg/mL, pH 3.8), NBT (1 mg/mL), Evans blue, or trypan blue solution was used to treat each selected leaf from both treatment and the controls at 25°C in darkness for 6 h, respectively. Ten plant leaves (biological replicates) were run for each treatment. Then, the dyed leaves were treated in 95% ethanol with boiled temperature to remove chlorophyll and placed on a light box to take photographs, as described by a previous study (Fryer et al., 2002).

### Statistical Analysis

Two-way analysis of variance was used to analyze the significant differences between the treatments and the controls. During the gene expression analysis, three biological replicates were run for each treatment; for avoiding the potential pipetting errors, three technical replicates were run for each gene. For physiological traits, each treatment was repeated for at least three times. *Post hoc* Duncan multiple-comparisons test was performed with significant levels of ^∗^*p* < 0.05, ^∗∗^*p* < 0.01, and ^∗∗∗^*p* < 0.001. All data were represented as means ± SD.

## Results

### Identification and Characterization of *HcTOE3*

**FIGURE 1:**
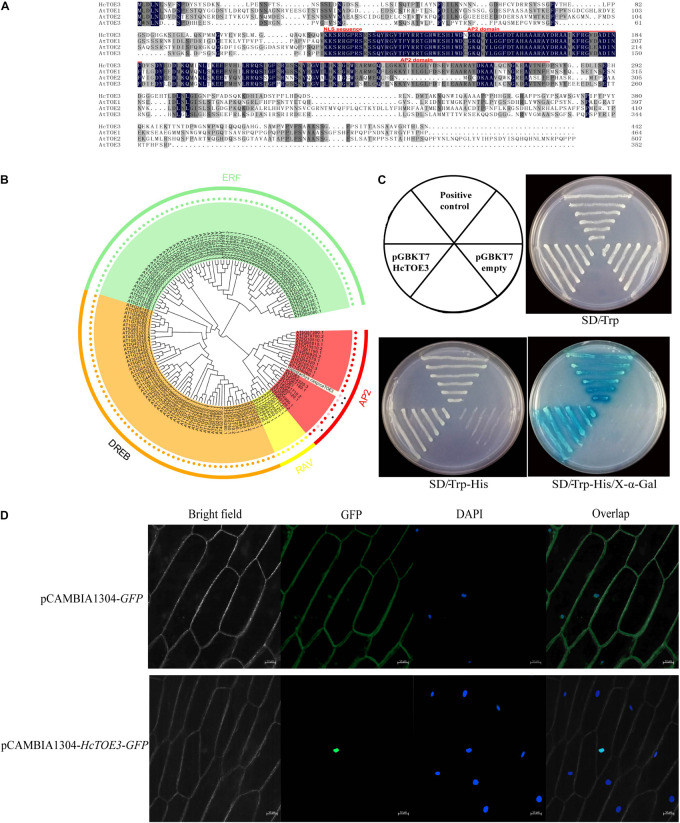
The analysis of the conserved domains **(A)**, phylogenetic relationship **(B)**, transcriptional activation of HcTOE3 **(C)**, and subcellular localization of HcTOE3 **(D)**.

In our previous work, *TOE3* was cloned from *H. caspica* by homologous cloning and RACE (Zhang et al., 2016). Based on the sequence analysis, there are a total of 1,329 bp in the *HcTOE3* ORF. *HcTOE3* gene encodes a 48.8-kDa protein containing 443 aa. By comparing with *Arabidopsis* TOE proteins, two AP2 DNA-BDs were found in the HcTOE3 protein, which are located at 130–186 and 222–279 aa, respectively. HcTOE3 protein also contains a nuclear localization signal (NLS) sequence that is located at 118–125 aa (Figure 1A). A phylogenetic tree containing both HcTOE3 and 150 *Arabidopsis* AP2/ERF TFs showed four distinct clades among these proteins. These four clades were AP2, RAV, ERF, and DREB clades comprising of 21, 7, 65, and 56 proteins, respectively. HcTOE3 was the closest to AtTOE3 protein; HcTOE3 was clustered into AP2 subfamily (Figure 1B).

### Transcriptional Activation Activity of HcTOE3

To ascertain whether HcTOE3 functions as a TF, transactivation assay was performed in yeast. As shown in Figure 1C, the positive control and yeast containing pGBKT7-*HcTOE3* gene divided quickly and formed the big biomass with blue color; however, no significant growth was observed in the negative control, and the yeast did not show the blue color. This suggests that HcTOE3 is activated and functions well in the yeast cells.

### Subcellular Localization of HcTOE3 Protein

Protein sequence alignment showed that HcTOE3 contained a conserved NLS domain. To localize where the HcTOE3 protein functions, the *HcTOE3* gene was linked to a *GFP* report gene and then transformed into onion epidermal cells. The green fluorescence was only observed in the nucleus (Figure 1D). This suggests that HcTOE3 is a nuclear-localized protein; after it is translated, it will be translocated into the nucleus.

### Expression Patterns of *HcTOE3* in *H. caspica* During Stress and ABA Treatments

**FIGURE 2:**
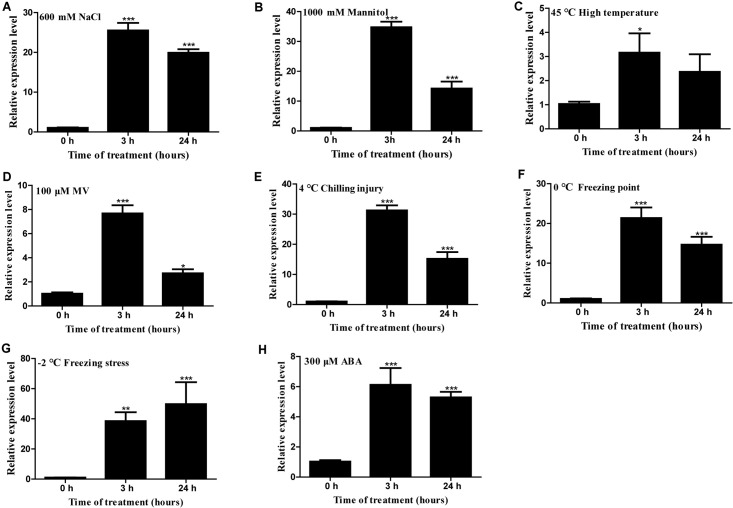
Expression profile of *H. caspica* assimilating branch *TOE3* gene under different abiotic stress and abscisic acid treatments: 600 mM NaCl **(A)**, 1,000 mM mannitol **(B)**, 45°C high temperature **(C)**, 100 μM MV **(D)**, 4°C chilling injury **(E)**, 0°C freezing point **(F)**, −2°C freezing stress **(G)**, 300 μM abscisic acid (ABA) **(H)**. *HcUBC10* served as reference gene, and the expression of *HcTOE3* gene in the control (0 h) was adjusted to 1. Data represented the average of three biological replicates. Significant differences at *p < 0.05, **p < 0.01, and ***p < 0.001.

Gene expression analysis showed that *HcTOE3* was strongly induced by NaCl, mannitol (simulated drought stress), heat (45°C), MV, chilling injury (4°C), freezing point (0°C), freezing stress (−2°C), and ABA (Figure 2). These results suggested that *HcTOE3* participated in abiotic stress responses and ABA-mediated exogenous hormone signal transduction pathways, and *HcTOE3* may be a rapid response gene in the early stage of stress response.

### Overexpression of *HcTOE3* Improves Freezing Tolerance in *Arabidopsis* by Enhancing Membrane Stabilization

**FIGURE 3:**
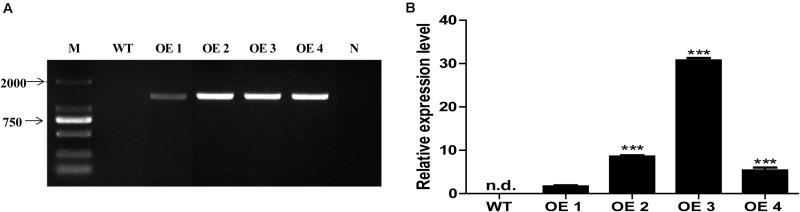
The identification and selection of overexpression *Arabidopsis*. **(A)** Genomic DNA PCR analysis of *HcTOE3* gene from WT and four transgenic lines. M, DNA marker; N, negative control using water as template. **(B)** Relative expression of *HcTOE3* in WT and four transgenic lines with *AtActin2* as an internal control using qRT-PCR. Data represented the average of three biological replicates. Significant differences were at ***p < 0.001.

To elucidate the functions of *HcTOE3* gene, *HcTOE3* was transferred into *Arabidopsis* by *Agrobacteria*-mediated floral dip transformation with CaMV 35S promoter. Four independent transgenic overexpression lines (OE, T2) were obtained, as confirmed by genomic DNA PCR and qRT-PCR analysis (Figure 3). Two independent homozygous T2 lines (OE2 and OE3) with the highly expressed *HcTOE3* were selected for testing plant tolerance to freezing stress.

**FIGURE 4:**
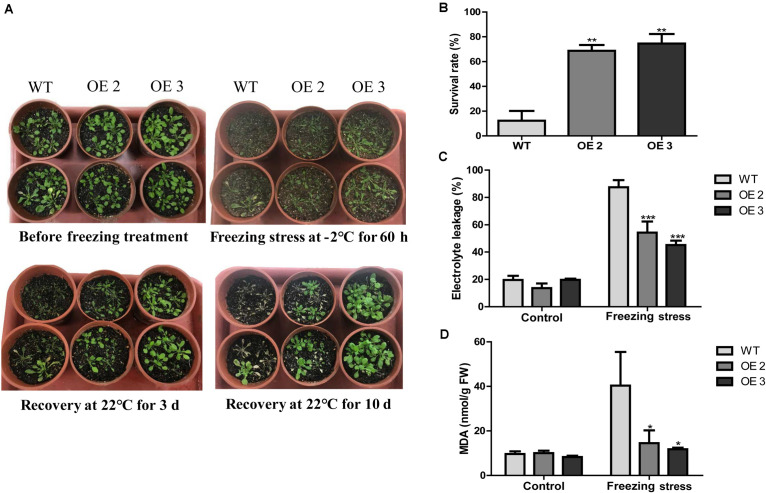
Overexpression of *HcTOE3* confers freezing tolerance in *Arabidopsis*. **(A)** Phenotype of the *HcTOE3-*overexpressing plants and the non-transgenic controls under freezing stress. **(B)** Survival rates were counted from 46 biological replicates. **(C)** Electrolyte leakage. **(D)** MDA content. Data represented the average of three biological replicates. Significant differences at *p < 0.05, **p < 0.01, and ***p < 0.001.

To examine the effects of *HcTOE3* transgenic lines on freezing tolerance condition, a freezing stress treatment was carried out with the T2 generation of homozygous transgenic *Arabidopsis* (OE2 and OE3) and the WT plants growing in pots under normal conditions for 4 weeks. As shown in Figure 4A, transgenic lines showed significantly enhanced freezing stressed tolerance than the controls. Under freezing treatment, the survival rate of *HcTOE3* transgenic lines was all greater (60%) compared to 20% for the WT (Figure 4B). Electrolyte leakage (EL) and MDA were inversely correlated with cell membrane stability and used as indicators to assess cell membrane damage (Bajji et al., 2002; Ashrafi et al., 2015). Here, all *HcTOE3-*overexpressing plants showed clearly lower EL values and MDA content than WT after freezing treatment (Figures 4C,D). These results indicated that expressing *HcTOE3* increase plant tolerance to freezing treatment.

### Overexpression of *HcTOE3* Enhanced the Antioxidant Capacity and Osmotic Adjustment Substance Content in Transgenic Plants Under Freezing Stress

**FIGURE 5:**
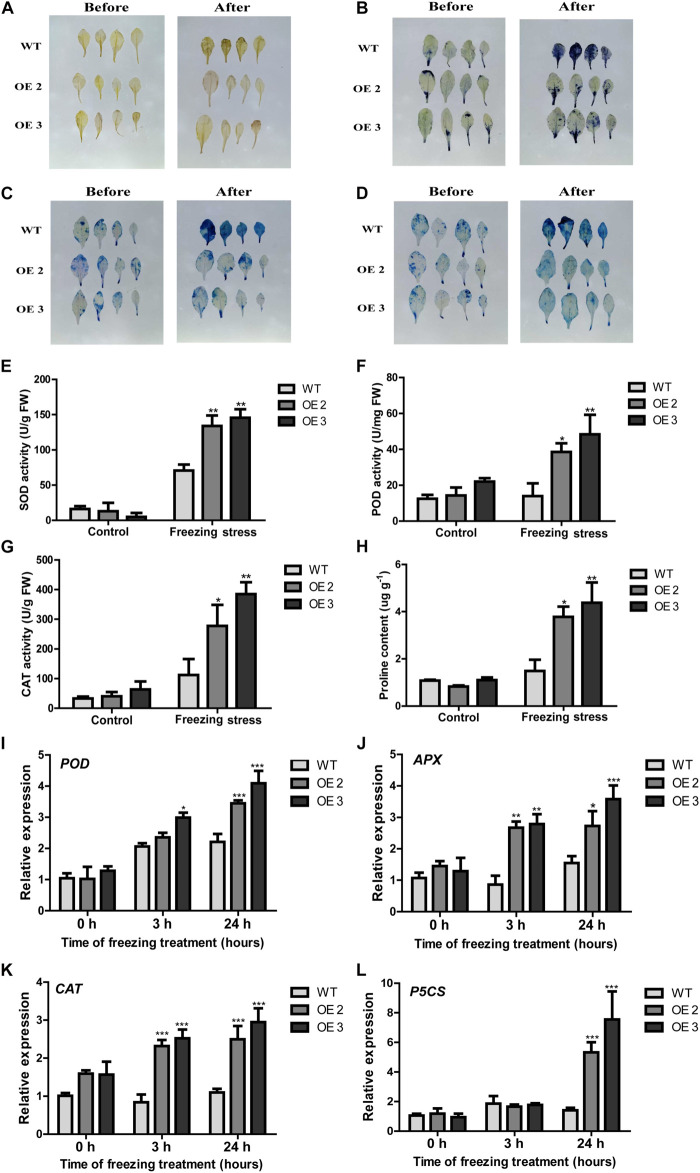
The performance of *HcTOE3* transgenic *Arabidopsis* under freezing treatment. **(A)** H_2_O_2_ detection in plant leaves by DAB staining. **(B)** O_2_^–^ detection in plant leaves by NBT staining. **(C**, **D)** Dead cell detection in plant leaves by Evans blue and trypan blue staining. **(E)** SOD activity. **(F)** POD activity. **(G)** CAT activity. **(H)** Proline content. **(I–K)** Expression levels of antioxidant enzyme genes, including *POD*, *APX*, and *CAT*. **(L)** Expression level of osmotic regulation gene *P5CS*. Data represented the average of three biological replicates. Significant differences at *p < 0.05, **p < 0.01, and ***p < 0.001.

It is well-known that unfavorable stress induces ROS accumulation (Dinakar et al., 2012). DAB and NBT staining were used to detect H_2_O_2_ and O_2_^–^ accumulation levels; trypan and Evans blue staining were used to examine the cell death in leaves from *HcTOE3* transgenic lines and WT under freezing stress. As shown in Figures 5A–D, less intensively brown and blue staining can be observed in the transgenic plants than those in the WT after freezing treatment, suggesting that *HcTOE3* transgenic plants had less accumulation of H_2_O_2_ and O_2_^–^ and less severe cell damage after suffering freezing stress.

To further explore the function of *HcTOE3* in enhancing plant tolerance to freezing stress, we examined physiological indicators in connection with ROS scavenging and osmotic adjustment substance contents in the plants during freezing stress treatment and the non-treated controls. For the controls, there were no difference on the activities of the antioxidant enzymes (SOD, CAT, and POD) between the transgenic plants and the WT controls. However, although freezing treatment increased the activities of all tested antioxidant enzymes in both transgenic plants and the controls, the activities were induced more in the transgenic plants than those in the WT controls (Figures 5E–G). Additionally, the accumulation of proline content was higher in the transgenic *Arabidopsis* plants after freezing stress (Figure 5H). To further confirm the above physiological data, we also examined the expression of the osmotic-related genes *P5CS* and the ROS detoxification-associated genes (*POD*, *CAT*, and *APX*). The qRT-PCR results demonstrated that freezing treatment induced the expression of all four tested genes more in the *Arabidopsis* OE plants than that in the non-transgenic controls (Figures 5I–L). All these evidence suggested that overexpressing *HcTOE3* enhances transgenic plant tolerance to freezing stress by increasing the contents of osmotic solutes and antioxidant enzyme activities.

### HcTOE3 TF Activates the Expression of Stress-Responsive Genes

**FIGURE 6:**
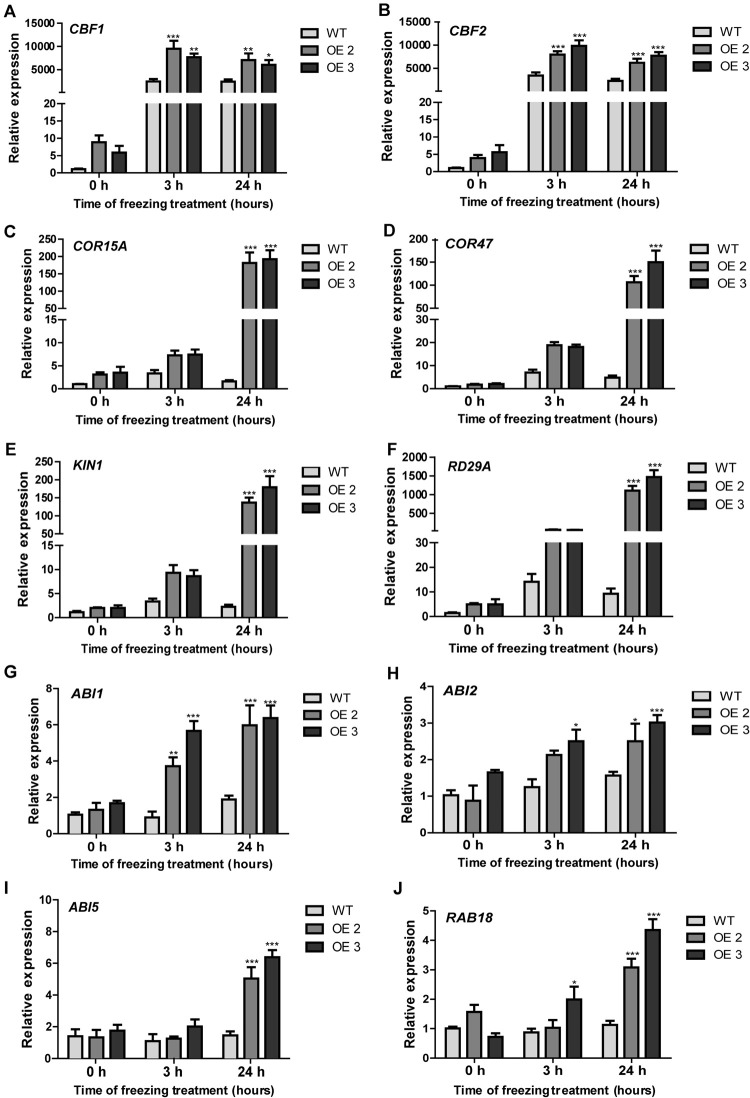
Expression patterns of cold-responsive and ABA-associated genes in the transgenic *Arabidopsis* and the non-transgenic plants under freezing stress treatment. **(A–F)** Expression profiles of cold responsive genes, including *CBF1*, *CBF2*, *COR15A*, *COR47*, *KIN1*, and *RD29A*. **(G–J)** Expression profiles of ABA-related genes, including *ABI1*, *ABI2*, *ABI5*, and *RAB18*. Data represented the average of three biological replicates. Significant differences at *p < 0.05, **p < 0.01, and ***p < 0.001.

HcTOE3 is a TF controlling the expression of other genes. To answer the question whether or not HcTOE3 induced the expression of low temperature–responsive genes and how they are related to each other. We detected the expression of several cold-associated genes, such as *CBF1* and *CBF2* and their associated genes, including *COR15A*, *COR47*, *KIN1*, and *RD29A*. During normal growth condition, there was no significant difference on the expression of six tested genes between the transgenic plants and the non-transgenic controls. However, under freezing treatment, the expression levels of the six tested genes were significantly higher in *HcTOE3*-overexpressing plants than those in the non-transgenic controls (Figures 6A–F). Additionally, we also examined the expression of genes associated with ABA signaling pathway, which included *ABI1*, *ABI2*, *ABI5*, and *RAB18*. The results indicated that freezing stress induced the expression of four ABA-responsive genes (Figures 6G–J). All of the above results suggested that HcTOE3 plays a critical role in enhancing plant tolerance to freezing stress potentially by regulating cold and ABA-responsive genes under freezing stress.

## Discussion

### *HcTOE3* Encodes an AP2/ERF TF Associated With Plant Response to Phytohormone ABA and Environmental Stresses

AP2/ERF TFs are one of the major TF gene families with a larger number of gene members. According to their gene structure and evolution relationship, AP2/ERF family can be divided into different subfamilies. Although different member of AP2/ERF may play different roles in plant growth and development, some members, such as ERF, DREB, and RAV, play an important function in plant response to various abiotic stresses (Xing et al., 2017; Zhao et al., 2019; Li et al., 2020; Lv et al., 2020). TOEs are AP2 TF family members, which inhibit CONSTANS and regulate flowering time by transmitting photoperiodic signal (Zhang et al., 2015). However, there is no report on the AP2 TFs in *H. caspica*. This study is the first report on the freezing tolerance identification of a novel TF gene *HcTOE3* from *H. caspica*. The HcTOE3 protein contains two typical AP2 domains belonging to AP2 subfamily and has a close relationship with AtTOE3 (Figures 1A,B). HcTOE3 exhibited transcriptional activation activity and localized in the nucleus (Figures 1C,D). In Lily, the expression of *LlDREB2B* was rapidly increased under cold, salt, mannitol, and heat treatment (Wu et al., 2018). Similarly in *H. caspica*, *HcTOE3* gene was strongly induced by salinity, mannitol (simulated drought), heat, MV, chilling injury, freezing point, freezing stress, and ABA treatments (Figure 2). These results indicated that *HcTOE3* may play significant roles in enhancing plant tolerance to abiotic stresses.

### Overexpressing *HcTOE3* Gene Enhanced Plant Tolerance to Freezing Stress

Under abiotic stresses, plants usually produce different ROS with a high level. As we know, ROS is a class of oxidative chemicals that damage plant cell membrane and cell structure, cause cell EL, and further inhibit plant growth and development (Caverzan et al., 2019). It was reported that low temperature significantly induced AP2/ERF gene expression in *Brassica napus* (Du et al., 2016). Overexpression of *AtERF102* and *AtERF103* demonstrated the important role of AP2/ERF gene in plant tolerance to cold stress potentially due to maintenance of electrolyte balance between inside and outside of the cells (Illgen et al., 2020). To study the function of *HcTOE3*, we obtained *HcTOE3*-overexpressing *Arabidopsis* (Figure 3), and the freezing tolerance of mature plants was examined; we found that overexpressing *HcTOE3* reduced EL in the transgenic plants; we also found MDA content was also decreased in the transgenic plants than that in the WT (Figures 4C,D). Our results agree with the previous results reported by Illgen et al. (2020). Overexpressing *HcTOE3* had less accumulation of O_2_^–^ and H_2_O_2_ in transgenic *Arabidopsis* plants than those in the WT by DAB and NBT staining (Figure 5). All the evidence demonstrated that overexpressing *HcTOE3* enhanced plant tolerance to freezing stress.

### The Freezing Tolerance of the Transgenic Plants of Overexpressed *HcTOE3* Was Related to Osmotic Adjustment, Antioxidative Competence, and Related Gene Expression

Freezing stress destroys water potential dispensation in plant cells, which further causes cell dehydration and even death (Pearce, 2001). Accumulation of proline and soluble proteins was associated with enhanced tolerance to freezing stress in plants (Kaplan and Guy, 2004; Kaplan et al., 2007). In our experiments, compared with WT plants, *HcTOE3* overexpressed plants showed higher proline content under freezing-stressed treatment (Figure 5H). *P5CS* is a crucial gene in the proline glutamate biosynthesis pathway, which increased proline accumulation and enhanced cold tolerance in several plants (Liu et al., 2020). After freezing treatment, the expression of *P5CS* was higher in transgenic plants than that in non-transgenic controls, which was consistent with proline content (Figure 5L). These results suggest that enhanced osmolytes may increase freezing tolerance in the *HcTOE3* OE plants.

Plants have evolved antioxidative systems to sustain ROS at low homeostasis levels. Plant enhanced their tolerance to various abiotic stresses by scavenging excessive ROS through antioxidant enzyme system (Chen et al., 2014; Ma et al., 2018). Here, we found that the activities of all tested antioxidant enzymes were increased in both transgenic and non-transgenic plants under freezing stress; however, the increased levels were higher in the transgenic plants than those in the non-transgenic plants (Figures 5E–G). *AtCAT*, *AtPOD*, and *AtAPX* encode CAT, POD, and ascorbate peroxidase (APX), respectively. They are thought to catalyze a lot of oxidative reactions by using H_2_O_2_ as an electron acceptor (Orvar and Ellis, 1995; Pasqualini et al., 2007). The transcriptional abundance of antioxidant genes was induced by freezing treatment in plants with overexpression of *HcTOE3* gene (Figures 5I–K). These results suggest that the freezing tolerance of *HcTOE3* transgenic *Arabidopsis* may be related to the enhanced antioxidative system.

There are several known pathways to mediate plant response to cold, of which the most classic one is the CBF pathway, which is associated with transcription regulators CBF1–3. Some targeted downstream genes of *CBF* have been identified, including *COR15A* and *COR47* (Wang et al., 2017). Our gene expression analysis showed that freezing treatment induced the expression of many low temperature–responsive genes, such as *CBF1*, *CBF2*, *COR15A*, *COR47*, *KIN1*, and *RD29A*, which were tested in this study. Freezing stress also induced the expression of the genes associated with ABA signaling pathway, which included *ABI1*, *ABI2*, *ABI5*, and *RAB18*. These results indicated that *HcTOE3* may depend on the CBF signaling pathway to control plant response to freezing stress. The ABA-dependent cold signaling pathway has long been considered to be mostly independent of the CBF pathway (Bolt et al., 2017). However, certain downstream genes, such as the TF MYB96, may integrate and activate both ABA and CBF pathways (Lee and Seo, 2015). Future work may be needed to investigate how HcTOE3 proteins are integrated into the widespread transcriptional networks that regulate plant response to cold stress. More research may also need to test the function of ABA regulation of HcTOE3 in the integrated freezing response pathway.

## Conclusion

**FIGURE 7:**
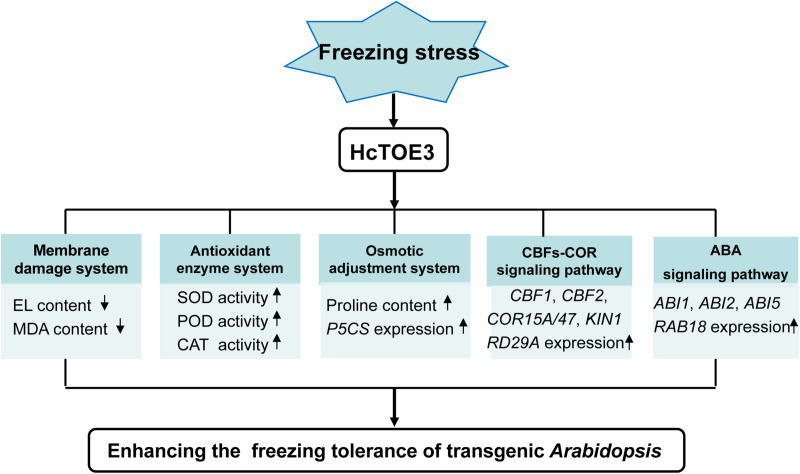
Model of HcTOE3 regulation of the freezing stress response in transgenic *Arabidopsis*. The model indicated that overexpression of *HcTOE3* up-regulates genes taking part in CBFs-COR signaling, ABA signaling, proline biosynthesis, and ROS scavenging. These genes lead to an increase in SOD, POD, CAT activities and proline content; a reduction ROS content and cell membrane peroxidation; and ultimately enhancing the freezing tolerance of *Arabidopsis*.

HcTOE3 is an AP2/ERF TF and belongs to the AP2 subfamily. AP2 subfamily, including TOE3, mainly plays a crucial role in plant growth and evolution. However, under abiotic stress, the function of HcTOE3 remains poorly understood. Transcription of *HcTOE3* in *H. caspica* was enhanced by environmental stresses, as well as exogenous plant hormone ABA treatments. Overexpression of *HcTOE3* resulted in elevated freezing tolerance of transgenic *Arabidopsis*. *HcTOE3* transgenic *Arabidopsis* reduced membrane lipid peroxidation and cell electrolyte extravasation. *HcTOE3* transgenic *Arabidopsis* plants also enhanced plant tolerance to freezing stress through increasing antioxidant enzyme activities and the contents of osmoregulation substances, such as proline. HcTOE3 mediates cold and ABA signal transduction by increasing the transcription level of stress-responsive genes, thereby improving plant tolerance to freezing stress in transgenic *Arabidopsis*. Our findings put forward a model to propose how HcTOE3 plays a significant role in enhancing freezing tolerance and provide valuable insight into the plant response to cold stress (Figure 7).

## Data Availability Statement

The original contributions presented in the study are included in the article/Supplementary Material, further inquiries can be directed to the corresponding author/s.

## Author Contributions

FY and YoZ designed the experiments, analyzed the data, and wrote the manuscript text. FY, JJ, PW, YuZ, and WL performed the experiments. All authors reviewed the manuscript.

## Conflict of Interest

The authors declare that the research was conducted in the absence of any commercial or financial relationships that could be construed as a potential conflict of interest.

## Footnotes

1 http://smart.embl-heidelberg.de/

2 https://www.predictprotein.org/

## Supplementary Material

Supplementary Table 1 The summary of the amino acid sequences from *Arabidopsis* AP2/ERF transcription factor members and HcTOE3 used to generate phylogenetic tree.

Supplementary Table 2 Primer sequences used in the present study.
