# Supplementary material for: The Halophyte Halostachys caspica AP2/ERF Transcription Factor HcTOE3 Positively Regulates Freezing Tolerance in Arabidopsis
Source: Front Plant Sci. 2021 May 13;12:638788. doi: 10.3389/fpls.2021.638788 (PMC8155596; doi:10.3389/fpls.2021.638788)
Supplement: Supplementary Table 2 — Primer sequences used in the present study. [file Table_2.docx]

| **Supplementary Table 2**  **Primer sequences used in the present study** | | |
| --- | --- | --- |
| **Primer name** | **Primer sequence (5’-3’)** | **Aim of amplifying genes with primers** |
| HcTOE3-Forward | ACGATGGAGGCGGCGAACATA | qRT-PCR for the expression of *HcTOE3* gene under various abiotic stress and ABA applyment in *H. caspica* |
| HcTOE3-Reverse | GAGGTGCTGCTGCTGCTTGAAT |  |
| HcUBC10-Forward | GATATACTAAAGGAACAGTGGAGCCC | As an internal reference gene of qRT-PCR in *H. caspica* |
| HcUBC10-Reverse | GACCATGGCATACTTCTGAGTCC |  |
| HcTOE3-Forward | GGTACCATGTTGGATCTTAATCT | To determine the integration and expression of *HcTOE3* in transgenic *Arabidopsis* |
| HcTOE3-Reverse | GGATCCTCAGTTAGATAAGTG |  |
| pGBKT7-HcTOE3-Forward | CGGGAATTCATGTTGGATCTTAATC | Vector construction for transcriptional activation in yeast cell |
| pGBKT7-HcTOE3-Reverse | CGCGGATCCGTTAGATAAGTGAGTTC |  |
| HcTOE3-GFP-Forward | GAAGATCTGTTGGATCTTAATCTAAGT | Vector construction for subcellular localization in onion cell |
| HcTOE3-GFP-Reverse | GGACTAGTGTTAGATAAGTGAGTTCGA |  |
| AtPOD-Forward | CCAAACTCTTCGTGGACTATGC | qRT-PCR of antioxidase genes including *POD*, *CAT*, *APX* in *Arabidopsis* |
| AtPOD-Reverse | AACTCTTGGTCGCTCTGGAT |  |
| AtCAT-Forward | GCAACTACCCCGAGTGGAAA |  |
| AtCAT-Forward | TGTTCAGAACCAAGCGACCA |  |
| AtAPX-Forward | CTCTGGGACGATGCCACAAG |  |
| AtAPX-Reverse | CTCGACCAAAGGACGGAAAA |  |
| AtP5CS-Forward | GGGACAAGTTGTGGATGGAGAC | qRT-PCR of proline synthetase (*P5CS*) in *Arabidopsis* |
| AtP5CS-Reverse | TGGTACAAACCTCAAGGAACAC |  |
| AtCBF1-Forward | GCATGTCTCAACTTCGCTGA | qRT-PCR of cold-responsive genes including *CBF1*, *CBF2*, *COR15A*, *COR47*, *KIN1* and *RD29A*  in *Arabidopsis* |
| AtCBF1-Reverse | ATCGTCTCCTCCATGTCCAG |  |
| AtCBF2-Forward | TGACGTGTCCTTATGGAGCTA |  |
| AtCBF2-Reverse | CTGCACTCAAAAACATTTGCA |  |
| AtCOR15A-Forward | TTCCACAGCGGAGCCAAGCA |  |
| AtCOR15A-Reverse | CGTCACCTTTAGCGGCGTAGAT |  |
| AtCOR47-Forward | CAGTGTCGGAGAGTGTGGTG |  |
| AtCOR47-Reverse | ACAGCTGGTGAATCCTCTGC |  |
| AtKIN1-Forward | ACCAACAAGAATGCCTTCCA |  |
| AtKIN1-Reverse | CCGCATCCGATACACTCTTT |  |
| AtRD29A-Forward | GCCGAGAAACTTCAGATTGG |  |
| AtRD29A-Reverse | CCATTCCTCCTCCTCCTTTC |  |
| AtABI1-Forward | CGTGTTTTCGGTGTTCTCGC | qRT-PCR of ABA signal transduction pathway genes including *ABI1*, *ABI2*, *ABI5* and *RAB18* in *Arabidopsis* |
| AtABI1-Reverse | AATCCGCTTCCTTGCCATCT |  |
| AtABI2-Forward | GGGCTCGTGTATTTGGTGTT |  |
| AtABI2-Reverse | GAGCCAAATCGCACACTTCT |  |
| AtABI5-Forward | ATGATCAAGAACCGCGAGTCTGC |  |
| AtABI5-Reverse | CGGTTGTGCCCTTGACTTCAAAC |  |
| AtRAB18-Forward | GAGCACCACGAGAAGAAGG |  |
| AtRAB18-Reverse | GCACAATACAACGACCGAATG |  |
| Atactin2-F | GGTAACATTGTGCTCAGTGGTGG | As an internal reference gene of qRT-PCR in *Arabidopsis* |
| Atactin2-R | AACGACCTTAATCTTCATGCTGC |  |
